# First clinical experiences of concurrent bleeding control and intracranial pressure monitoring using a hybrid emergency room system in patients with multiple injuries

**DOI:** 10.1186/s13017-018-0218-x

**Published:** 2018-11-29

**Authors:** Takahiro Kinoshita, Kazuma Yamakawa, Jumpei Yoshimura, Atsushi Watanabe, Yosuke Matsumura, Kaori Ito, Hiroyuki Ohbe, Kei Hayashida, Shigeki Kushimoto, Junichi Matsumoto, Satoshi Fujimi, on behalf of the Japanese Association for Hybrid Emergency Room System (JA-HERS) Scientific Promotion Committee

**Affiliations:** 1 Division of Trauma and Surgical Critical Care, Osaka General Medical Center, 3-1-56 Bandai-Higashi, Sumiyoshi-ku, Osaka, 558-8558 Japan; 2 Department of Emergency and Critical Care Medicine, Chiba University Graduate School of Medicine, 1-8-1 Inohana, Chuo-ku, Chiba, 260-0856 Japan; 3 Department of Emergency Medicine, Division of Acute Care Surgery, Teikyo University School of Medicine, 2-11-1 Kaga, Itabashi-ku, Tokyo, 173-8606 Japan; 4 Division of Emergency and Critical Care Medicine, Tohoku University Graduate School of Medicine, 1-1 Seiryo-cho, Aoba-ku, Sendai, 980-8574 Japan; 5 Department of Emergency and Critical Care Medicine, School of Medicine, Keio University, 35 Shinanomachi, Shinjuku-ku, Tokyo, 160-8582 Japan; 6 Department of Emergency and Critical Care Medicine, St. Marianna University School of Medicine, 2-16-1 Sugao, Miyamae-ku, Kawasaki, 216-8511 Japan

**Keywords:** Concurrent treatment, Functional outcome, HERS, Mortality, Polytrauma

## Abstract

**Background:**

The outcomes of multiple injury patients with concomitant torso hemorrhage and traumatic brain injury (TBI) are very poor. The hybrid emergency room system (HERS) is a trauma management system designed to complete resuscitation, computed tomography (CT), surgery, angioembolization, and intracranial pressure (ICP) monitoring all in one trauma resuscitation room without patient transfer. We aimed to review the outcomes of polytrauma patients who underwent concurrent bleeding control and ICP monitoring using the HERS.

**Methods:**

In this retrospective observational study, we enrolled patients who underwent concurrent bleeding control and ICP monitoring using the HERS between August 2011 and June 2018. Initial data on vital signs, Injury Severity Score (ISS), probability of survival (Ps) calculated by the Trauma and Injury Severity Score (TRISS), intervention type, 28-day mortality, and Extended Glasgow Outcome Scale at 6 months after injury were collected. Continuous variables were expressed as the median (25th and 75th percentiles) and categorical variables as numbers (%).

**Results:**

Ten patients were included in the analysis. The injury severity of the patients was as high as an ISS of 58 (50–64) and TRISS Ps of 0.15 (0.02–0.36). Seven of the 10 (70%) patients had hemodynamic instability within 30 min from arrival. The recorded durations from arrival to events were CT examination 9 (6–16) min, bleeding control procedure 29 (22–42) min, and neurosurgical intervention 39 (31–53) min. Four of the 10 patients (40%) survived to discharge, and two of them (20%) were able to live independently at 6 months after injury.

**Conclusions:**

The concurrent performance of bleeding control procedure and ICP monitoring would be feasible in HERS settings among polytrauma patients with exsanguinating hemorrhage and TBI.

**Electronic supplementary material:**

The online version of this article (10.1186/s13017-018-0218-x) contains supplementary material, which is available to authorized users.

## Background

Advanced Trauma Life Support guidelines have long been used in the initial management of patients with suspicions of severe injury [1]. These guidelines emphasize limiting the use of computed tomography (CT) as a detailed secondary survey examination only in hemodynamically stable patients without an apparent indication for emergency surgery. Thus, CT scanning for hemodynamically unstable patients is not practical until the completion of emergency bleeding control surgery even in cases with a decreased level of consciousness; traumatic brain injury (TBI) in conjunction with torso hemorrhage is often left untreated or even unidentified. This notion is based on the concern that CT is a time-consuming procedure that involves the considerable risk of deterioration during CT scanning itself and/or patient transfer to CT suites. Although the emergence of multidetector-row CT has dramatically reduced the time required for CT scanning [2], the time delay to definitive surgery associated with the transfer of patients to a room with CT equipment remains unacceptable in those with hemodynamic instability.

The hybrid emergency room system (HERS) is a novel trauma management system that is potentially suitable for the evaluation and care of severe multiple injury patients [3–5]. The key component of this system is the trauma resuscitation room that is designed for the completion of all the examinations and treatments in a single place and which includes angio-CT equipment, a ventilator, a vital sign monitor, ultrasonography equipment, and surgical, neurosurgical, and interventional radiology (IR) instruments. Our novel trauma workflow using the HERS enables the performance of whole-body CT even in hemodynamically unstable patients with hemodynamic resuscitation under monitoring [4]. Furthermore, it is possible to concurrently perform bleeding control procedures including thoracotomy, laparotomy, preperitoneal pelvic packing, and endovascular intervention and surgical intracranial pressure (ICP) monitoring without patient transfer, based on CT findings. This progress allows for the advancement of titrating neuroprotective therapies formulated according to the CT findings, ICP, and cerebral perfusion pressure (CPP) in the bleeding control intervention phases.

Previously, we reported that a novel trauma workflow using the HERS was significantly associated with reduced mortality in severe blunt trauma patients [4] and improved functional outcomes in patients with severe TBI [5]. In the present study, we aimed to review a series of patients with hemorrhagic torso injury in conjunction with TBI, who underwent concurrent bleeding control and ICP monitoring.

## Methods

### Study population

In this retrospective observational study, we enrolled consecutive blunt polytrauma patients who underwent concurrent bleeding control and ICP monitoring using the HERS in the Osaka General Medical Center from August 2011 to June 2018. We defined concurrent treatment as a combination of interventions in which the recorded durations of bleeding control (through direct surgery or endovascular embolization) and ICP monitoring and/or ventriculostomy overlapped, whichever started first. Patients with cardiopulmonary arrest on arrival, who underwent bleeding control and ICP monitoring separately and who were transferred from other hospitals, were excluded from the analysis. The study was approved by the Institutional Review Board of the Osaka General Medical Center (#30-S07-001). The board waived the need for informed consent, as this was a retrospective study.

### Treatment strategy for polytrauma patients

In the Osaka General Medical Center, an original approach was formulated for polytrauma patients with concomitant exsanguinating hemorrhage and TBI. First, airway and breathing abnormalities were evaluated and fixed at the earliest after patients’ arrival. We fundamentally performed whole-body CT to detect all life-threatening injuries including torso hemorrhages and TBIs even in patients with shock since CT could be conducted within approximately 10 min from arrival through the use of angio-CT equipment, with hemodynamic resuscitation under continuous monitoring [4]. If patients’ vital signs were too unstable for 10-min CT scanning, we proceeded to more aggressive resuscitation including blood transfusion, resuscitative thoracotomy, and resuscitative endovascular balloon occlusion of the aorta (REBOA). When REBOA was used prior to CT examination, a self-propelled C-arm was manipulated to the patient table for the insertion of the REBOA catheter with a fluoroscopic guide. The aorta was occluded partially rather than fully, targeting a systolic blood pressure (BP) of 90–100 mmHg for the preservation of cerebral blood flow and also the prevention of possible intracranial hemorrhage expansion.

**Fig. 1:**
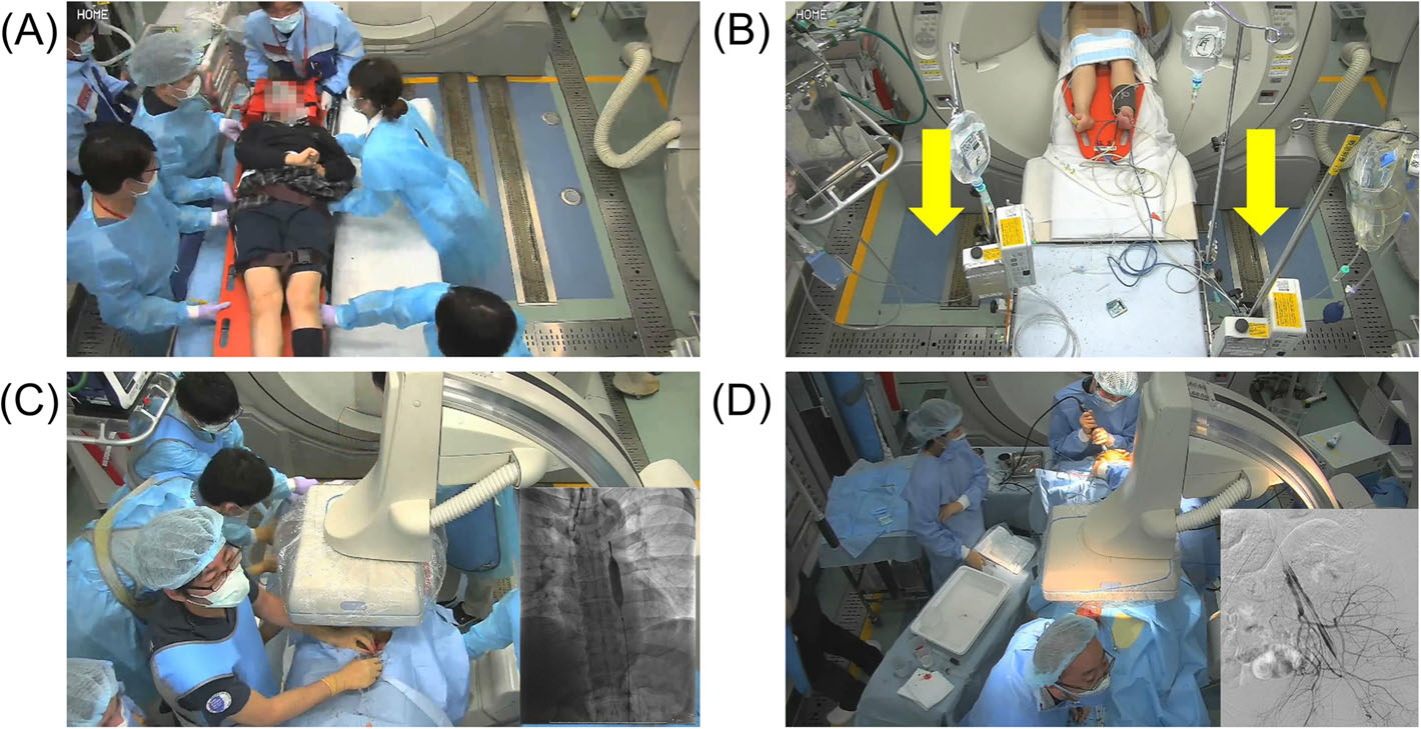
Photographs of the concurrent treatments performed using the HERS. **a** All doctors and nurses wear radiation protection products before patients’ arrival. Patients are directly accommodated in the hybrid emergency room on arrival. Chest X-ray, pelvic X-ray, and FAST are not performed routinely preceding CT scanning. **b** After intravenous access is achieved, whole-body CT examination is performed as soon as possible. Head and neck CT are routinely performed without contrast and chest, abdominal, and pelvic CT with contrast. The gantry of the CT scanner moves instead of the patients’ table during CT scanning. **c** A REBOA catheter is inserted with fluoroscopic guidance to avoid complications. The balloon inflation volume is controlled to maintain a systolic BP of 90–100 mmHg before ICP measurement and to preserve a CPP ≥ 60 mmHg after ICP monitoring. **d** Bleeding control procedures and ICP monitoring and/or ventriculostomy are performed simultaneously if active torso bleeding and significant intracranial lesion are detected by CT. Neurosurgeons make sure not to interfere with surgical procedures and IR since priority is always given to hemostasis. BP, blood pressure; CPP, cerebral perfusion pressure; CT, computed tomography; FAST, focused assessment with sonography for trauma; HERS, hybrid emergency room system; ICP, intracranial pressure; IR, interventional radiology; REBOA, resuscitative endovascular balloon occlusion of the aorta

When life-threatening torso hemorrhage and TBI were observed on CT, we embarked upon the simultaneous treatment of both injuries. An attending trauma surgeon decided on the performance of direct surgery (thoracotomy, laparotomy, and preperitoneal pelvic packing), angioembolization, or both based on CT findings. At the same time, an attending neurosurgeon initiated intracranial surgery for ICP monitoring and/or ventriculostomy without waiting for the completion of the bleeding control procedure (Fig. 1). If a space-occupying lesion with a midline shift was identified in the CT images, the neurosurgeons created two burr holes in the frontal bone and attempted to evacuate epidural or subdural hematomas to the greatest extent possible. Discussions were held with trauma surgeons for the provision of the best available neurocritical care for each patient guided by early ICP monitoring. More specifically, neuroprotective treatments to maintain a CPP ≥ 60 mmHg were selected from among 30° head elevation, sedation, analgesia, hyperosmolar treatment, and the administration of fluid, blood, and inotropes, with attention to hemodynamic status and the anatomical sites of injuries. After the completion of concurrent treatment, we routinely re-evaluated the brain CT and determined if craniotomy was to be added in an operating room or if continued critical/neurocritical care was to be provided in an intensive care unit.

### Data collection

Initial data on vital signs (Glasgow Coma Scale total score, BP, and heart rate [HR]) were collected for the first measurement. The lowest BP within 30 min from arrival was also extracted from patients’ records. Unstable vital signs were defined as hypotension (systolic BP ≤ 90 mmHg), tachycardia (HR ≥ 120 beats per minutes), or deterioration of BP (systolic BP ≤ 90 mmHg) within 30 min after arrival, indicating the high risk associated with transferring patients to a CT room in the conventional trauma workflow. The results of laboratory tests including platelet counts, prothrombin time-international normalized ratio, and activated partial thromboplastin time were collected. The Abbreviated Injury Scale of each body region was recorded to measure the Injury Severity Score (ISS). Findings from the initial CT examination were categorized based on Marshall CT classification: diffuse injury I, II, III, IV, and non-evacuated mass lesion [6]. The probability of survival (Ps) was calculated using the Trauma and Injury Severity Score (TRISS) (coefficients were b0 − 1.2470, b1 0.9544, b2 − 0.0768, and b3 − 1.9052) [7]. Data on the type of surgery, IR, and neurosurgical intervention conducted in the hybrid emergency room were recorded. The intervals from patient arrival to CT examination, the bleeding control procedure, and ICP measurement were described. Data on 24-h mortality were extracted from patient records, and the Extended Glasgow Outcome Scale at 6 months post injury was evaluated from records, referral documents, or telephone interviews.

### Descriptive statistics

Continuous variables were expressed as the median (25th and 75th percentiles) as the data were not normally distributed. Categorical variables were described as numbers (%). Descriptive statistics were performed with R software packages (version 3.1.0; R Foundation for Statistical Computing, Vienna, Austria).

## Results

**Fig. 2:**
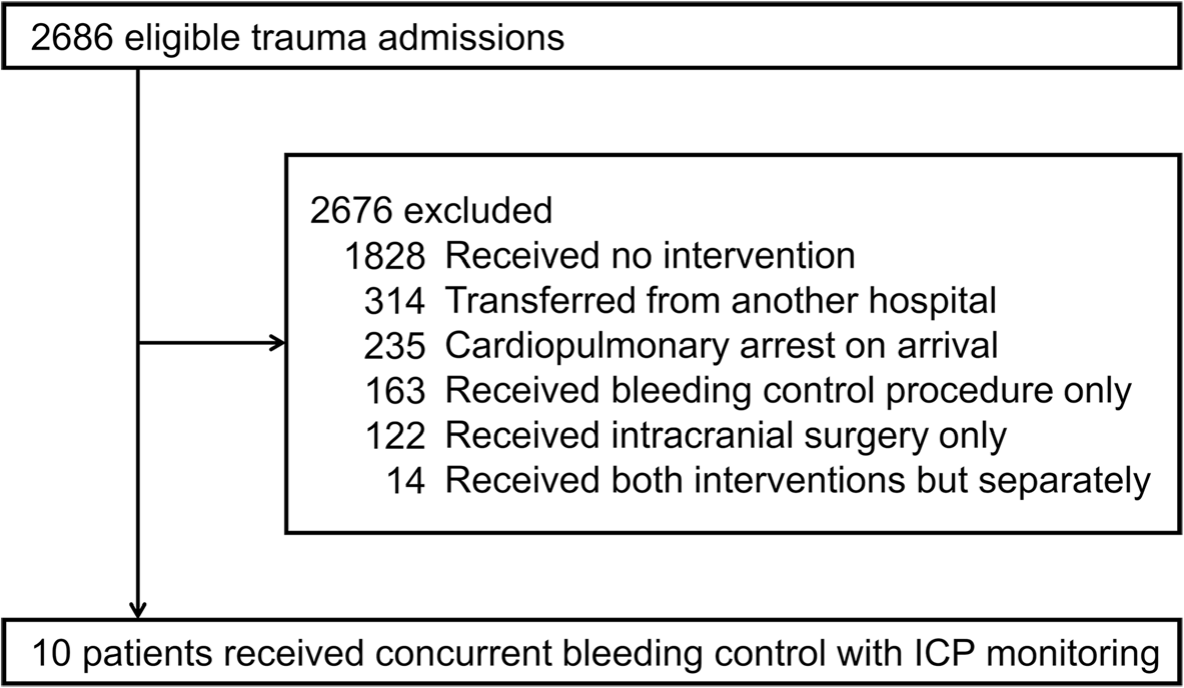
Patient enrollment flow diagram. ICP, intracranial pressure

Of 2686 eligible patients admitted for trauma in the Osaka General Medical Center during the 8-year study period, 10 were identified for the reception of concurrent bleeding control and ICP monitoring (Fig. 2). Table 1 shows the patients’ baseline characteristics. The age of the patients was 43 (30–60) years. The injury severity of the patients was extremely high, as indicated by the ISS 58 (50–64) and TRISS Ps 0.15 (0.02–0.36). Most of the included patients had high risks for the performance of whole-body CT in conventional trauma settings, since all but three had hemorrhagic shock status on arrival or their condition deteriorated within 30 min of arrival.

**Table 1:** Baseline characteristics of the multiple injury patients who underwent concurrent treatment

Case	Age (years)	Sex	GCS total score	Initial BP (mmHg)	HR (bpm)	Lowest BP within 30 min (mmHg)	Platelet count (× 10^4^/L)	PT-INR	aPTT (s)	Marshall CT classification	ISS	Ps
1	52	Female	6	183/137	140	66/52	13.9	1.5	65	Diffuse injury IV	59	0.37
2	62	Female	3	154/88	56	74/40	12.3	1.7	> 200	Non-evacuated mass lesion	66	0.01
3	72	Female	10	155/116	113	112/45	17.4	1.3	56	Non-evacuated mass lesion	59	0.15
4	36	Male	3	142/59	133	88/42	12.8	1.7	63	Diffuse injury III	66	0.07
5	49	Male	8	126/78	97	107/65	16.0	1.2	35	Non-evacuated mass lesion	45	0.65
6	34	Male	4	194/147	116	52/20	21.2	1.4	54	Non-evacuated mass lesion	50	0.36
7	29	Female	3	46/25	151	30/20	18.3	1.7	74	Non-evacuated mass lesion	57	0.02
8	18	Female	8	110/80	155	105/79	17.0	1.5	52	Diffuse injury II	50	0.49
9	29	Female	3	60/30	117	58/37	15.2	1.4	44	Non-evacuated mass lesion	66	0.02
10	74	Male	6	175/111	102	97/70	17.2	1.1	50	Non-evacuated mass lesion	45	0.25

Ps was calculated using the Trauma and Injury Severity score

*aPTT* activated partial thromboplastin time, *BP* blood pressure, *CT* computed tomography, *GCS* Glasgow Coma Scale, *HR* heart rate, *ISS* Injury Severity Score, *Ps* probability of survival, *PT-INR* prothrombin time-international normalized ratio

Table 2 shows the contents and timelines of interventions and patients’ outcome. The recorded durations from patient arrival to events were CT examination, 9 (6–16) min; bleeding control procedure, 29 (22–42) min; and neurosurgical intervention, 39 (31–53) min. ICP monitoring was used not only to guide patients’ neurocritical care but also to prepare for further neurosurgical interventions, with concerns of expanded intracranial hemorrhage during the bleeding control procedure in one patient (No. 5), in whom the ICP was elevated to 40 mmHg in the midst of an endovascular intervention, suggesting the indication for emergency craniotomy. Two of the patients (Nos. 7 and 9) underwent REBOA for hemodynamic stabilization before CT scanning. In these patients, partial REBOA was applied to avoid an excessive increase in the ICP and intracranial hemorrhage exacerbations. Specifically, we attempted to control the balloon inflation volume to maintain a systolic BP of 90–100 mmHg before ICP measurement and to preserve a CPP ≥ 60 mmHg after ICP monitoring (see video, Additional file 1: Digital Content 1, which demonstrates the trauma workflow in one patient with concomitant torso exsanguinating hemorrhage and severe TBI [No. 7]).

**Table 2:** Contents and timelines of interventions and patients’ outcomes

Case	Time to CT scan (min)	Bleeding control procedure	Time to bleeding control procedure (min)	Intracranial surgery	Time to intracranial surgery (min)	24-h mortality	GOS-E at 6 months
1	5	Angioembolization (IIA)	54	ICP measurement, burr hole drainage	53	Dead	D
2	7	Angioembolization (ICA, IIA)	28	ICP measurement, craniotomy	28	Dead	D
3	10	Angioembolization (IIA)	43	ICP measurement	51	Dead	D
4	6	PPP, angioembolization (IIA)	21	ICP measurement, ventricular drainage	29	Alive	VS
5	9	Angioembolization (ICA)	30	ICP measurement, craniotomy	35	Alive	Upper SD
6	18	Angioembolization (ICA, SA, IIA)	25	ICP measurement, burr hole drainage	26	Alive	D
7	24	REBOA, angioembolization (HA, IIA), PPP	18	ICP measurement, burr hole drainage	56	Alive	Lower MD
8	5	Angioembolization (ITA)	47	ICP measurement	43	Alive	Lower MD
9	18	REBOA, PPP, angioembolization (IIA)	13	ICP measurement, burr hole drainage	35	Dead	D
10	9	PPP, angioembolization (IIA)	40	ICP measurement, craniotomy	59	Alive	D

*CT* computed tomography, *D* death, *GOS-E* Extended Glasgow Outcome Scale, *HA* hepatic artery, *ICA* intercostal artery, *ICP* intracranial pressure, *IIA* internal iliac artery, *ITA* internal thoracic artery, *MD* moderate disability, *PPP* preperitoneal pelvic packing, *REBOA* resuscitative endovascular balloon occlusion of the aorta, *SA* splenic artery, *SD* severe disability, *VS* vegetative state

Four of the 10 patients (40%) survived to discharge and two of them (Nos. 7, 8) were able to live independently at 6 months after injury. None of the patients was considered to have perished from exsanguination since the CPP decreased to less than 20 mmHg before hemodynamic collapse in all patients who did not survive.

## Discussions

In this study, we reported the clinical courses of multiple injury patients who underwent the simultaneous performance of a bleeding control procedure and rigorous ICP/CPP-oriented neuroprotective management. We confirmed that concurrent treatment for the exsanguination of torso hemorrhage and TBI was feasible in HERS settings. Furthermore, four of our patients had survival to discharge and two had favorable functional outcomes at 6 months post injury. Notably, we were able to successfully treat two patients with the most severe injuries, who had TRISS Ps as low as 0.02 and 0.07. To the best of our knowledge, this is the first study to report on the outcomes of patients who received concurrent surgery/angioembolization with neurocritical care following ICP monitoring in HERS settings.

Exsanguination and TBI are reciprocally harmful, creating a vicious circle; hemorrhage causes hypotensive secondary brain injury that increases the mortality associated with TBI [8–13] while TBI leads to coagulopathy that precludes hemorrhagic injuries from hemostasis [14–17]. These relations lead to extremely poor outcomes in polytrauma patients with active thoracic, abdominal, and retroperitoneal hemorrhage and severe TBI. For example, a recent post hoc study of a randomized control trial reported that patients with concomitant TBI and hemorrhagic shock had significantly higher mortality, received higher amounts of transfusion, and developed a higher proportion of respiratory complications than patients without TBI and hemorrhagic shock [18]. Nevertheless, there is no consensus regarding an effective method that can be used to reduce the associated mortality and improve the functional outcomes of these patients. Concurrent treatment is potentially beneficial in the provision of appropriate neurocritical care based on ICP/CPP without the need to wait for the completion of bleeding control procedures. However, the performance of this treatment is basically impractical in hemorrhagic shock patients because the evaluation of TBI by CT is considered a prerequisite for neurosurgical intervention even though patients with an unstable condition require bleeding control before CT.

The HERS was designed to overcome this problem. The installation of a CT scanner in a trauma resuscitation room enabled the performance of CT, which is indispensable for TBI management even in hemodynamically unstable patients. Furthermore, the introduction of surgical and neurosurgical instruments as well as a C-arm in this room allowed for the simultaneous provision of damage control surgery, endovascular intervention including REBOA and angioembolization, and invasive ICP measurement and/or ventriculostomy. That is to say, we were able to perform concurrent treatment for multiple injury patients by combining the functions of a CT scan room with the recently reported-upon hybrid operating room [19]. We believe the HERS, which was invented with high aspiration to provide resuscitation, CT examination, damage control surgery, IR, and ICP monitoring “all in one room”, is an ideal trauma suite setting for the realization of this innovative intervention.

The present study has several limitations. First, we could not find a well-matched comparative group due to the retrospective nature of the study design. It was impossible to demonstrate an existence of TBI in patients in whom we suspected multiple injuries within the conventional period, because these patients frequently died during or soon after bleeding control procedure without CT scanning. Therefore, the effectiveness of concurrent treatment, using the HERS, on patient outcomes should be investigated in a prospective study. Second, we did not determine the indication for concurrent treatment. We provided it when an attending surgeon and neurosurgeon who participated in trauma resuscitation decided that both a bleeding control procedure and ICP monitoring were required and feasible based on CT findings. The objective indication for concurrent treatment should be investigated in a future study. Third, the management strategy other than concurrent treatment may have changed during the study period since the 8-year observational period was relatively long. Finally, we did not provide this treatment to penetrating trauma patients. As the efficacy of whole-body CT scanning in penetrating trauma is still not well-explored, we could not discuss the effect of the present treatment in this population.

## Conclusions

We described the first clinical case series of patients who underwent concurrent bleeding control and ICP monitoring using the HERS. The HERS could be a breakthrough in the treatment of the most severe polytrauma cases requiring both bleeding control and neurosurgical procedures. The effect of the concurrent treatment on patients’ clinical outcomes should be investigated in a prospective interventional study.

## Additional file

Additional file 1: Digital Content 1. Video of the trauma workflow in a patient with concomitant torso hemorrhage and TBI. The video shows the trauma workflow using the HERS in one patient with concomitant torso hemorrhage and TBI (Case: No. 7). HERS, hybrid emergency room system; TBI, traumatic brain injury (MP4 19062 kb)

## Abbreviations

BP: Blood pressure
CPP: Cerebral perfusion pressure
CT: Computed tomography
HERS: Hybrid emergency room system
HR: Heart rate
ICP: Intracranial pressure
IR: Interventional radiology
ISS: Injury severity score
Ps: Probability of survival
REBOA: Resuscitative endovascular balloon occlusion of the aorta
TBI: Traumatic brain injury
TRISS: Trauma and Injury Severity Score
